# Clinical and epidemiological characteristics of hospitalized children with enterovirus infection: a retrospective study from 2016 to 2025 at Beijing children’s hospital

**DOI:** 10.1186/s12879-026-12988-2

**Published:** 2026-03-07

**Authors:** Fei Li, Yong Sun, Luci Huang, Ruiting Yang, Jianing Yao, Yue Cui, Qiuping Li, Xiangpeng Chen, Zhengde Xie

**Affiliations:** Beijing Key Laboratory of Core Technologies for the Prevention and Treatment of Emerging Infectious Diseases in Children, Key Laboratory of Major Diseases in Children, Ministry of Education, National Clinical Research Center for Respiratory Diseases, Research Unit of Critical Infection in Children, Chinese Academy of Medical Sciences, 2019RU016, Laboratory of Infection and Virology, Beijing Pediatric Research Institute, Beijing Children’s Hospital, Capital Medical University, National Center for Children’s Health, Beijing, 100045 China

**Keywords:** Enterovirus, Clinical characteristics, Epidemiology, Children

## Abstract

**Background:**

Enteroviruses (EVs) are important pathogens that cause neurological and systemic infections in children. Currently, only hand, foot, and mouth disease (HFMD) is monitored in China, indicating a lack of systematic surveillance for other EV-related diseases. Additionally, long-term epidemiological data on pediatric EV infections in Beijing are scarce. The aim of this study was to describe the clinical and epidemiological characteristics of children with EV infection in Beijing over a ten-year period.

**Methods:**

In this retrospective study, all hospitalized patients who underwent EV RNA detection using a fluorescence‑based real‑time PCR assay between July 2016 and July 2025 were included. Demographic, clinical, and laboratory information was retrieved from the hospital’s electronic medical record system.

**Results:**

A total of 8,426 patients was included in this study, and 236 cases (2.80%) tested positive for EVs. Notably, males exhibited a higher detection rate (3.14%, 160/4,940) than females (2.18%, 76/3,486). Moreover, children aged 3 to < 6 months (5.26%, 13/247) and those aged 3 to < 7 years (4.51%, 56/1243) had higher detection rates than other age groups. In terms of seasonal patterns, EV infections demonstrated distinct peak in summer and autumn. Among the 205 patients whose clinical information was complete, viral encephalitis (VE) was identified as the most predominant disease (55.12%, 113/205), particularly among children aged 3 to < 6 years (39.82%, 45/113) and those aged 28 days to < 3 months (10.62%, 12/113). This was followed by myocardial injury/myocarditis (23.41%, 48/205), pneumonia (22.93%, 47/205), hepatic dysfunction (14.63%, 30/205), multiple organ dysfunction syndrome (MODS) (8.78%, 18/205), HFMD (7.80%, 16/205), herpangina (3.90%, 8/205), and arrhythmia (3.41%, 7/205). In this study, 98.05% of the patients had a favorable prognosis, with a mortality rate of 1.95% (4/204).

**Conclusions:**

Diseases related to EV infection are among the important reasons for hospitalization in children, among which the top three diseases are encephalitis/meningitis, myocarditis/myocardial damage, and pneumonia. EV infection mainly affects infants and young children under 7 years of age, with the peak incidence in summer and autumn seasons.

**Clinical trial number:**

Not applicable.

**Supplementary Information:**

The online version contains supplementary material available at 10.1186/s12879-026-12988-2.

## Introduction

Enteroviruses (EVs) are small RNA viruses belonging to the genus Enterovirus within the family *Picornaviridae*. Human-infecting EVs are currently classified into four species (*alphacoxsackie*, *betacoxsackie*, *coxsackiepol* and *deconjuncti*) [1]. These viruses are primarily transmitted through the fecal oral route or via respiratory secretions, particularly in crowded settings or environments involving close contact. EVs cause a wide range of clinical manifestations, ranging from asymptomatic infections, which account for nearly half of all cases, to severe illnesses such as myocarditis and sepsis [2, 3]. Common clinical syndromes include hand, foot, and mouth disease (HFMD), herpangina, and acute hemorrhagic conjunctivitis [2]. Infants and immunocompromised individuals are particularly susceptible to severe complications.

In recent years, surveillance across various countries and regions has identified EVs as the primary pathogens responsible for viral encephalitis (VE) and viral meningitis (VM) in children. In the United States, the annual incidence of VM is estimated at to be 8–10 per 100,000 people [4, 5], and approximately 58% of VM cases among infants and young children are attributable to EV infections [6, 7]. With the global elimination eradication of poliomyelitis through vaccination, nonpolio EVs have emerged as the leading causes of central nervous system (CNS) infections [8, 9]. The main causative agents include enterovirus A71 (EV‑A71), coxsackievirus A6 (CV‑A6), coxsackievirus B1 (CV‑B1), echovirus 6 (E6), and other serotypes, as well as enterovirus D68 (EV‑D68) [10]. These viruses can cause severe neurological manifestations, including VE, VM, acute flaccid paralysis (AFP), and acute flaccid myelitis (AFM). Globally, the pediatric disease burden related to EV infections remains considerable [8, 10, 11]. Current vaccination strategies primarily target EV‑A serotypes (e.g., EV‑A71, CV-A6, CV-A10 and CV-A16), while effective vaccines and antiviral therapies for most other EV types are still unavailable. Consequently, the management of EV infections continues to rely chiefly on supportive care.

The World Health Organization (WHO) currently oversees the global poliovirus surveillance network but has not yet established a dedicated international system for nonpolio EV surveillance. By comparison, the United States initiated the National Enterovirus Surveillance System (NESS) in the 1960s [12]. Across Europe, most countries have developed national laboratory-based surveillance programs to systematically monitor the epidemiological trends of EV infections [13–15]. In the Asia-Pacific region, the Asia-Pacific Network for Enterovirus Surveillance (APNES) was established in Cambodia, Malaysia, Vietnam, and Taiwan, China [16]. However, the network’s effectiveness remains constrained by its limited geographic coverage. In China, a nationwide laboratory surveillance network for HFMD has been gradually developed in recent years [17–19]. A systematic, laboratory-based surveillance network for other EV-associated diseases has yet to be established. The aim of this study is to summarize and analyze the clinical and epidemiological characteristics of hospitalized children with EV infections in Beijing over the past decade, to inform the development of more effective prevention and control strategies.

## Materials and methods

### Study design and participants

This retrospective study included hospitalized patients who underwent EV RNA testing at Beijing Children’s Hospital, Capital Medical University, from July 1, 2016, to July 31, 2025. Participants were analyzed according to season: spring (March-May), summer (June-August), autumn (September-November), and winter (December-February of the following year). Cases were categorized by year, covering a span of ten years (2016–2025). For age classification, the following groups were established: <28 days, 28 days to < 1 year, 1 to < 3 years, 3 to < 7 years, and 7 to < 18 years. The cohort aged 28 days to < 1 year was subdivided into three categories: 28 days to < 3 months, 3 months to < 6 months, and 6 months to < 12 months.

### Inclusion and exclusion criteria

The inclusion criteria were as follows: from July 1, 2016, to July 31, 2025, all types of samples (rectal swab, throat swab, blood, and cerebrospinal fluid) subjected to EV RNA detection in the infectious diseases and virology laboratory at Beijing Children’s Hospital. The exclusion criterion was the repeated submission of the same type of sample from the same patient within the same week.

Criteria for diagnosing EV-positive cases: a positive result in sterile specimens, such as blood or cerebrospinal fluid, confirms the presence of the pathogen. For nonsterile samples, such as throat swabs and rectal swabs, EV infection is diagnosed when these specimens return positive results and other pathogenic infections are excluded through clinical evaluation.

### Specimen collection and EV detection

Trained medical personnel were responsible for collecting children’s rectal swabs, throat swabs, cerebrospinal fluid (CSF), and blood samples, which were immediately transported under low-temperature conditions to the laboratory. Rectal and throat swabs were collected using viral sampling tubes, CSF was collected in sterile collection tubes, and blood samples were collected in EDTA-anticoagulated tubes and centrifuged to obtain plasma for testing.

Total RNA/DNA from the samples was extracted using viral nucleic acid extraction kit (magnetic bead method, SDK60104) (Bioperfectus Technologies Co., Ltd., Jiangsu, China). EV RNA detection was performed using an EV nucleic acid detection kit (PCR-fluorescence probe method) (DaAn Gene Co., Ltd., Guangzhou, China).

### Data collection

Demographic and clinical data, including age, sex, admission date, length of stay, diagnosis, microbiological test results, laboratory examinations (such as CSF and routine blood tests), and imaging examinations (including cranial ultrasound, CT, and MRI), were obtained from the hospital’s electronic medical record system database.

### Statistical analysis

Continuous variables are expressed as the mean ± standard deviation (SD), with group comparisons conducted using t-tests for variables following a normal distribution. For nonnormally distributed variables, data are reported as medians (interquartile ranges, IQRs), and nonparametric tests were employed. Categorical variables are presented as counts (%), with group comparisons performed using chi-square tests or Fisher’s exact probability method as appropriate. Multivariate logistic regression analysis was performed to identify independent risk factors for enterovirus infection. Data analysis was conducted using Python version 3.13. A significance level of *P* < 0.05 was established.

## Results

### Demographic characteristics of EV-positive cases

A total of 8,426 patients were included in this study and the detection rate of EVs was 2.80% (236/8,426). Most patients were from the neonatal ward (29.52%), neurology ward (25.32%), and infectious diseases ward (15.81%), followed by the pediatric intensive care unit (9.53%), neonatal intensive care unit (6.15%), hematology ward (6.15%), and emergency intensive care unit (2.54%). Cases from all other departments contributed less than 1% of the total.

**Fig. 1:**
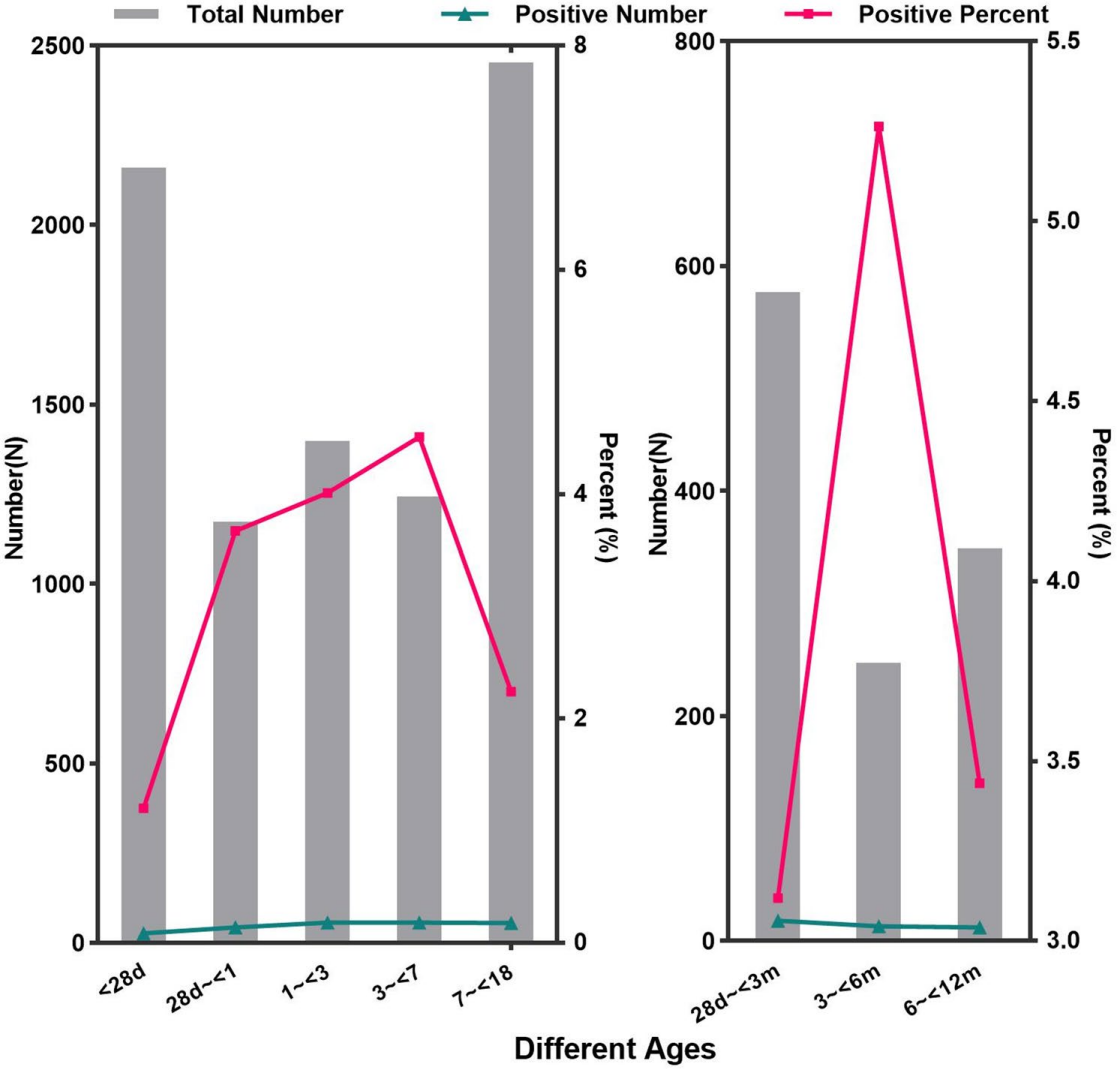
Age distribution of EV-positive patients (*n* = 236)

In the 236 patients, the positive detection rate was significantly higher in males (3.14%, 160/4940) than in females (2.18%, 76/3486) (χ^2^ = 8.009, *P* = 0.000). Moreover, positive detection rates differed significantly among the various age groups (χ^2^ = 71.683, *P* = 0.000) **(**Fig. 1**)**. The highest detection rate was observed in children aged 3 to < 7 years at 4.51% (56/1243), followed by those aged 1 to < 3 years at 4.01% (56/1398), 28 days to < 1 years at 3.67% (43/1173), 7 to < 18 years at 2.24% (55/2452) and < 28 days at 1.20% (26/2160). Among infants under one- year, notable differences in detection rates were recorded as follows (χ^2^ = 26.777, *P* = 0.000): infants younger than 28 days accounted for 1.20% (26/2160), those aged 28 days to < 3 months accounted for 3.12% (18/577), those aged 3 to < 6 months accounted for 5.26% (13/247), and those aged 6 to < 12 months accounted for 3.44% (12/349). Supplementary Figure S1 provides detailed age distribution information.

### Seasonal distributions of EV-positive cases

**Fig. 2:**
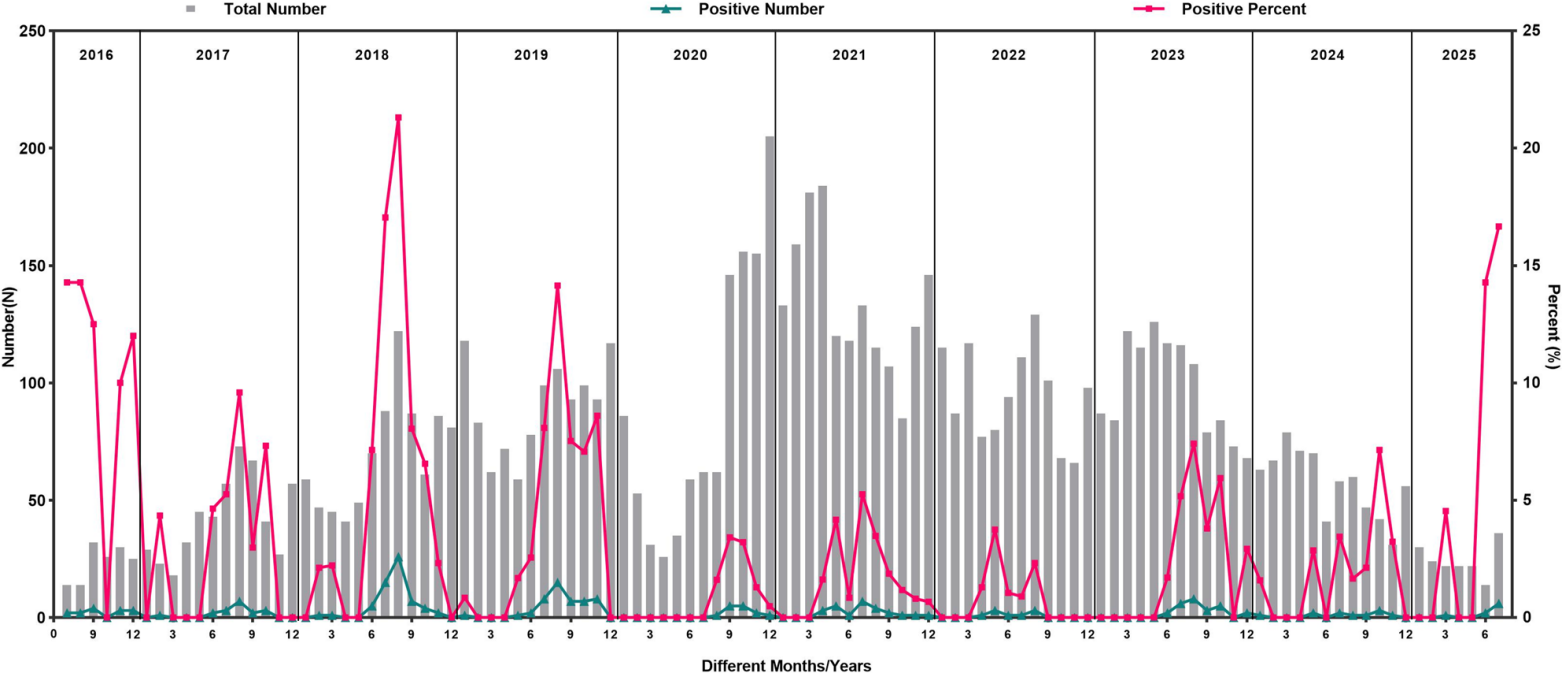
Distribution of EV-positive cases from 2016 to 2025

Seasonal analysis indicated that the highest detection rate occurred in summer at 6.01% (132/2197), followed by autumn at 3.61% (76/2106), spring at 0.88% (17/1923), and winter at 0.50% (11/2200) (χ^2^ = 156.800, *P* = 0.000). Before 2020, there was a notable peak in August and a smaller peak from January to March **(**Fig. 2**)**. However, in 2020, the primary peak shifted to September, and a minor peak was absent. From 2021 to 2025, the peak continued to increase but remained lower than pre-2020 levels. The peak began to return to normal levels in 2023, accompanied by the reemergence of a minor peak after 2024. The annual detection rates of EV RNA varied (χ^2^ = 126.479, *P* = 0.000), with detection rates decreasing from 2018 (7.30%) to 2022 (0.79%). The yearly detection rates were as follows: 2019 (4.54%), 2017 (3.52%), 2023 (2.21%), 2024 (1.61%), 2021 (1.56%), 2020 (1.30%), and 2022 (0.79%). Supplementary Figure S2 and Table S1 provide sample distribution information. Multivariate logistic regression analysis was further conducted and confirmed that gender, age, and season are independent risk factors for EV infection (Supplementary Table S2).

### Clinical characteristics and diseases of EV-positive patients

Among the 236 patients, complete clinical information was collected for 205 patients (86.86%), with a mean length of stay (LOS) of 12.89 ± 15.19 days. Among the children, 187 (91.22%) presented with fever, while 35 (17.07%) experienced convulsions. A complete cranial imaging study was conducted in 114 patients (55.61%), revealing abnormalities in 47 patients (41.23%).

**Fig. 3:**
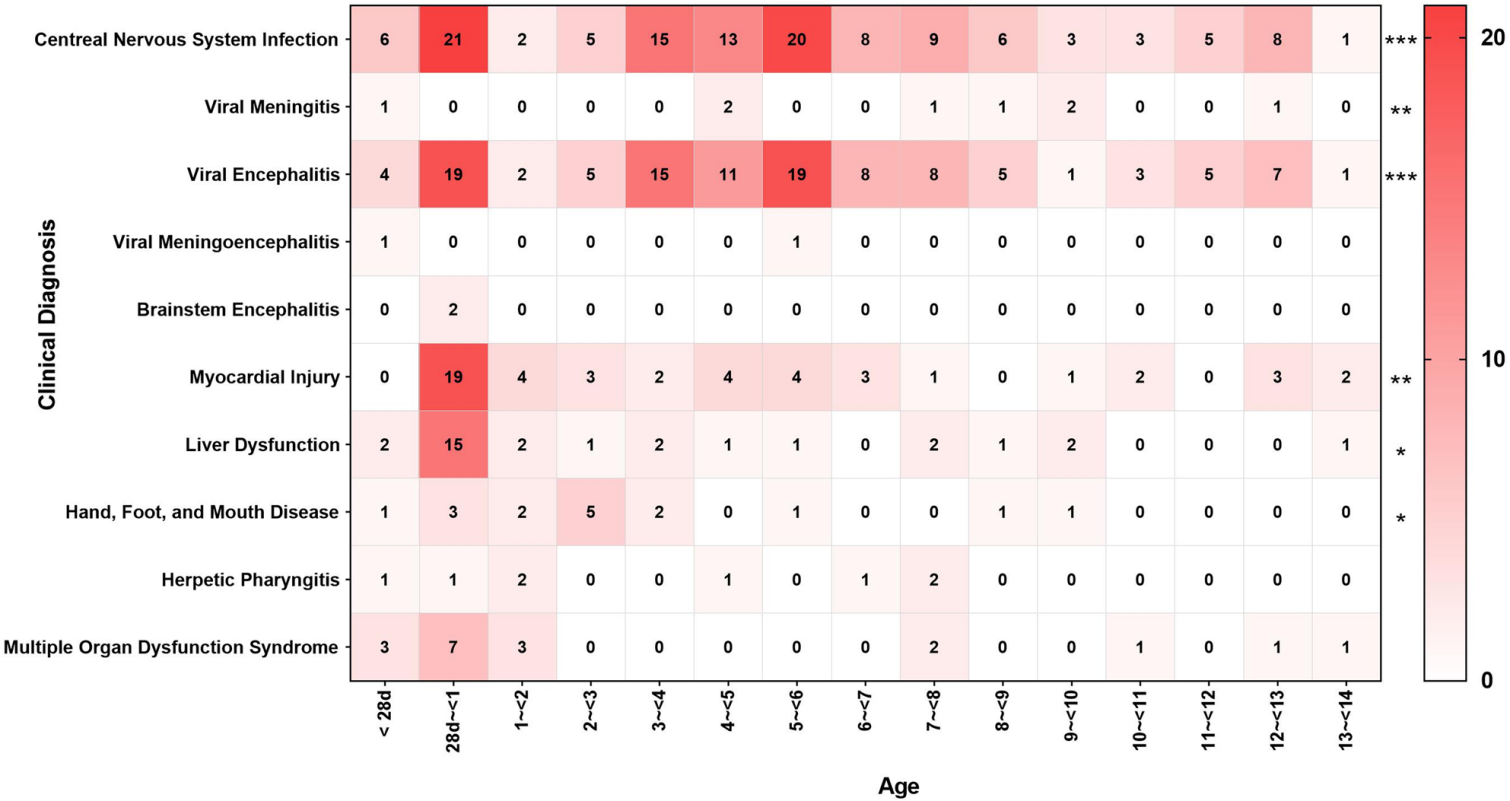
Disease distribution among children of different ages in EV-positive Cases (*N* = 205)

The clinical diagnoses varied widely among the 205 children **(**Table 1**)**. CNS infections accounted for 60.98% (125 cases) and were primarily classified as VE (55.12%, 113 cases), followed by VM (3.90%, 8 cases), viral meningoencephalitis (0.98%, 2 cases), and brainstem encephalitis (0.98%, 2 cases). The detection rates of VE varied significantly across different age groups (χ^2^ = 50.104, *P* = 0.002). Additional diagnoses included myocardial injury (23.41%, 48 cases), with myocarditis detected in 8 cases. Hepatic dysfunction occurred in 14.63% (30 patients), and Hand, foot, and mouth disease (HFMD) constituted 7.80% (16 cases) **(**Fig. 3**)**. Additionally, pneumonia was reported in 22.93% (47 cases) of patients, and multiple organ dysfunction syndrome (MODS) was reported in 8.78% (18 cases) of patients. Moreover, there were no differences in the clinical diagnoses between sexes (all *P* > 0.05). Among those children, infants aged 28 days to < 1 year represented a particularly vulnerable population **(**Fig. 3**)**. Notably, infants aged 28 days to < 1 year demonstrated a disproportionately elevated prevalence of severe diagnoses, particularly in myocardial injury (39.58%, 19/48), hepatic dysfunction (50.00%, 15/30), and MODS (38.89%, 7/18). Within the CNS infection, VE accounted for 16.81% (19/113 cases), with 63.16% (12/19) occurring in infants aged 28 days to < 3 months. Myocarditis was predominantly observed in the 28 days to < 3 months subgroup (47.37%, 9/19 cases). Hepatic dysfunction was predominantly concentrated in infants aged 28 days to < 6 months (86.67%, 13/15 cases). MODS manifestations were primarily distributed across the 6 months to < 12 months age range (71.43%, 5/7 cases).

**Table 1:** Disease spectrum of EV-positive patients (*N* = 205)

Clinical Diagnosis		Number (*n*)	Percent (%)
**Central Nervous System Diseases**			
	Central Nervous System Infection	125	60.98
	*Viral Meningitis*	8	3.90
	*Viral Encephalitis*	113	55.12
	*Viral Meningoencephalitis*	2	0.98
	*Brainstem Encephalitis*	2	0.98
**Respiratory System Diseases**			
	Bronchitis	16	7.80
	Pneumonia	47	22.93
**Hematologic Diseases**			
	Cytopenia	4	1.95
**Cardiovascular Diseases**			
	Myocardial Injury/Myocarditis	48	23.41
	Arrhythmia	7	3.41
**Digestive System Diseases**			
	Hepatic Dysfunction	30	14.63
	Neonatal Pathological Jaundice	12	5.85
**Skin and mucosal diseases**			
	Hand, Foot, and Mouth Disease	16	7.80
	Herpangina	8	3.90
**Others**			
	Multiple Organ Dysfunction Syndrome	18	8.78
	Sepsis	8	3.90
	Shock	6	2.93
	Septicemia	4	1.95
	Hemophagocytic Syndrome	2	0.98

### Clinical outcomes and prognosis

The majority of patients (98.05%, 201/205) recovered and were discharged, with 4 patients (1.95%, 4/205) died; of these, 3 deaths were attributed to MODS, and 1 death was attributed to brainstem encephalitis (Table 2). All four patients were male and under 1 year of age. The LOS ranged from 1 day to a maximum of 35 days.

**Table 2:** Clinical Information of Death Cases (*N* = 4)

Num.	Year	LOS	EV Positive Sample Types	Clinical Diagnosis
1	2016	33	rectal swab	MODS, Shock, Brainstem Encephalitis, CNS Infection, Hand, Foot, and Mouth Disease, Pneumonia
2	2018	12	CSF	Brainstem Encephalitis, CNS Infection, Myocardial Injury, Refractory Status Epilepticus, Pneumonia
3	2019	1	serum	MODS, Sepsis, Shock, Neonatal Pathological Jaundice, Neonatal Hypoglycemia
4	2024	35	rectal swab	MODS (Cerebral Dysfunction and Respiratory Failure)

LOS: length of stay, days; CSF: cerebrospinal fluid; MODS: multiple organ dysfunction syndrome; CNS: central nervous system

## Discussion

This retrospective study provides an overview of the clinical and epidemiological characteristics of EV infections in children hospitalized at Beijing Children’s Hospital over the past ten years. The overall detection rate of EVs was 2.80%. Notably, the highest detection rates occurred among males and children aged 3 to < 6 months, as well as those 3 to < 7 years. EV infections demonstrated distinct peaks during the summer and autumn months; however, NPIs related to SARS-CoV-2 disrupted the typical seasonal pattern. VE accounted for the largest proportion of clinical manifestations among EV-positive patients, particularly among children aged 3 to < 7 years and those aged 28 days to < 3 months. Four deaths occurred, with a mortality rate of 1.95%. This study provides valuable insights into the epidemiology of EV infections and highlights the urgent need for enhanced surveillance of severe cases, especially in younger hospitalized children.

This study revealed differences in EV-positive patients based on season, sex, and age. First, the positive detection rate among males was higher than that among females, which is consistent with the findings of Sun et al. in Hangzhou [20]. Liu et al. reported that from 2008 to 2014 in mainland China, compared with females, male children had a higher risk of infection in terms of both fatal and nonfatal cases of HFMD [21]. This may be attributed to biological factors and social behavior patterns, potentially increasing the exposure of males to pathogens and their susceptibility to infection. Additionally, the highest detection rate for EV positivity occurred among children aged 3 to < 6 months (5.26%) and those aged 3 to < 7 years (4.51%). Sun et al. reported that the rate of EV-positivity increased with age, from 0.99% among infants younger than one month to 8.42% among children aged 5 ~ ≤ 7 years, with the highest positivity observed in this age group [20]. This variation could be partially explained by our finer age stratification among younger infants, which revealed an earlier peak in EV positivity at 3 to < 6 months that may not have been captured in previous studies with broader age categories. Moreover, Shi et al. conducted a seroepidemiologic study on CV-A6 and EV-A71 infections involving 55,176 participants from 13 countries [22]. Their findings revealed that the antibody seropositivity rates for EV-A71 and CV-A6 were 36.3% and 55.8%, respectively, among children aged 0–5 years and 62% and 72%, respectively, among individuals aged 5 years and older [22]. These results indicate that children under five may be more susceptible to EV infections, highlighting the need for targeted preventive measures in this age group. Overall, the findings highlight the importance of sex and age in the context of EV infections, indicating that public health strategies should be tailored to target high-risk populations with appropriate interventions.

EV infections showed distinct summer-autumn peaks during 2017–2020 and re-emerged with similar patterns from 2023 to 2025. In contrast, a different pattern emerged from 2021 to 2022, with a significant particularly peak observed in spring. The summer peak returned to normal levels beginning in 2023, and a minor reemerged peak after 2024. Throughout the pandemic, the Chinese government implemented community-based preventive measures to curb viral transmission [23]. After nonpharmaceutical interventions (NPIs) were lifted, the number of EV infection cases resurged, which is consistent with findings from EV circulation surveillance in the United States, Europe and Taiwan, China [15, 24, 25]. Previous studies have demonstrated that NPIs exhibit the strongest protective effects during autumn and school summer vacations, while high average temperature and high relative humidity are significantly correlated with the incidence of HFMD, indicating that environmental and behavioral factors indeed affect the seasonal prevalence of EV [26, 27]. Furthermore, multiple studies have confirmed that NPIs implemented in response to SARS-CoV-2 have altered the seasonal patterns of various viruses, including respiratory syncytial virus (RSV), human metapneumovirus (HMPV) and adenovirus (ADV), while the seasonal impact on EVs appears to be delayed [28–30]. For example, Perez et al. reported that during the 2020–2021 season, the overall proportion of seasonal respiratory viruses was lower than that in previous seasons, except for human parainfluenza viruses (HPIV types 1–3) and rhinovirus (RV)/EV [29]. Our research also revealed that NPIs affected the seasonal prevalence of EVs, not in 2020 but in 2021, which was possibly due to the relatively stringent implementation of NPIs in 2021 and the gradual return of this effect to a normal seasonal pattern by 2023. Sun et al. (2019–2023) reported a year-to-year distribution of EV infections in children from Hangzhou that was largely consistent with our findings [20]. While our study revealed a reduced EV detection rate in 2020, the seasonal pattern persisted, whereas Sun et al. reported no clear seasonal epidemic in that year [20]. Therefore, this study highlights the need for ongoing surveillance of EV trends to promptly identify and address potential public health risks, particularly in light of the observed seasonal variations and the impact of concurrent viral infections, which may complicate disease management and outbreak response strategies.

Some studies conducted in India, Kuwait, and various European countries have reported EV detection rates ranging from 21 to 22% among encephalitis patients in endemic regions [31–33]. In China, the detection rate of EVs among VE patients in Hebei Province ranged from 7.32% to 16.50% between 2019 and 2023 [34, 35], while rate of EV positivity among clinical samples from febrile children in Hangzhou from 2010 to 2021 was 25.82% [36]. Our study revealed that VE accounted for 55.12% of EV‑positive cases. This higher proportion may be attributed to study design, which specifically included all nucleic acid-confirmed (PCR) EV‑positive cases rather than patients selected based on particular clinical symptoms or diagnostic criteria. Most children with EV infection (e.g., HFMD or herpangina) are treated as outpatients rather than being hospitalized, and only severe cases (such as severe HFMD, VE, and viral myocarditis) require hospitalization and EV testing in inpatient wards. Furthermore, this distribution explains the high proportion of VE among EV-positive inpatients. Notably, the diagnosis rate of VE among children aged 3 to < 7 years has reached 60% to 90%, in contrast to the 16% to 66% rate among children under 1 year of age, which aligns with the results of other regions in China [34, 37]. This age-related difference may be attributed to more frequent exposure in group settings and clearer clinical manifestations among older children [3, 10]. However, within the infant group, a notably high VE positivity rate of 66.67% was observed in those aged 28 days to < 3 months, indicating that increased vigilance is warranted in clinical practice. This heightened susceptibility may be attributed to the developmental immaturity of the infant immune system and the progressive decline of maternally acquired antibodies during this critical developmental window, collectively rendering young infants particularly vulnerable to EV neurotropism. In summary, this study underscores the importance of age-stratified risk assessment in managing EV infections and associated diseases in children. Enhanced surveillance and timely diagnosis of high-risk age groups are essential to mitigate disease burden and inform public health interventions.

EV infections present a broad spectrum of diseases that are primarily managed in outpatient settings. Severe cases may require hospitalization and can result in long-term sequelae or even death. In this study, we observed that most hospitalized children had a favorable prognosis, although a minority experienced fatal outcomes, mainly from MODS and brainstem encephalitis. EV-A71 is the most neurotropic EV after poliovirus, with approximately 30% of infected patients developing HFMD [38, 39]. Our findings revealed that hospitalized patients primarily had VE, with a low incidence of HFMD, and that all patients with HFMD were discharged in good condition. Moreover, an outbreak of E18 in the NICU in Qingdao, Shandong Province in 2019, resulted in septic-like symptoms in ten infants, all of whom improved without fatalities [40]. However, among the children diagnosed with MODS in this study, the mortality rate was 22.22% (4/18), underscoring the need for early detection and aggressive management. Overall, while most children with EV infections fare well, the risk of severe complications remains significant. Therefore, these findings highlight the importance of ongoing research and effective clinical management to improve patient outcomes.

This study has several limitations. First, this was a single-center study conducted in Beijing, which may restrict the generalizability of the findings. Second, the analysis focused exclusively on hospitalized children; however, the majority of EV infections are managed in outpatient settings, with only a minority of children requiring hospitalization due to more severe illness. Third, the study did not conduct further laboratory typing of the EVs, as different EV types may present distinct clinical pathogenesis profiles.

In conclusion, this study provides important insights into the clinical and epidemiological characteristics of hospitalized children in Beijing from 2016 to 2025. The results showed that diseases related to EV infection is an important cause of hospitalization in children, among which the top three diseases are encephalitis/meningitis, myocarditis/myocardial damage, and pneumonia; EV infection mainly affects infants and young children under 7 years of age, with the peak incidence in the summer and autumn seasons. Furthermore, this study clarifies the spectrum and clinical burden of EV-associated diseases in the hospitalized pediatric population and highlight the importance of recognizing EV infection in these key clinical syndromes to improve diagnosis and guide clinical management and public health strategies.

## Supplementary Information

Below is the link to the electronic supplementary material.

Supplementary Material 1

## Data Availability

Data is provided within the manuscript.

## Abbreviations

EVs: Enteroviruses
HFMD: Hand, foot, and mouth disease
MODS: Multiple organ dysfunction syndrome
VE: Viral encephalitis
VM: Viral meningitis
CNS: Central nervous system
EV-A71: Enterovirus A71
CV-A6: Coxsackievirus A6
CV-B1: Coxsackievirus B1
E6: Echovirus 6
EV-D68: Enterovirus D68
AFP: Acute flaccid paralysis
AFM: Acute flaccid myelitis
WHO: World Health Organization
NESS: National enterovirus surveillance system
APNES: Asia-pacific network for enterovirus surveillance
CSF: Cerebrospinal fluid
LOS: Length of stay
NPIs: Nonpharmaceutical interventions
RSV: Respiratory syncytial virus
HMPV: Human metapneumovirus
ADV: Adenovirus
RV: Rhinovirus
