# Supplementary material for: Clinical and epidemiological characteristics of hospitalized children with enterovirus infection: a retrospective study from 2016 to 2025 at Beijing children’s hospital
Source: BMC Infect Dis. 2026 Mar 7;26:760. doi: 10.1186/s12879-026-12988-2 (PMC13081482; doi:10.1186/s12879-026-12988-2)
Supplement: Supplementary file 1 — Supplementary Material 1 [file 12879_2026_12988_MOESM1_ESM.docx]

**
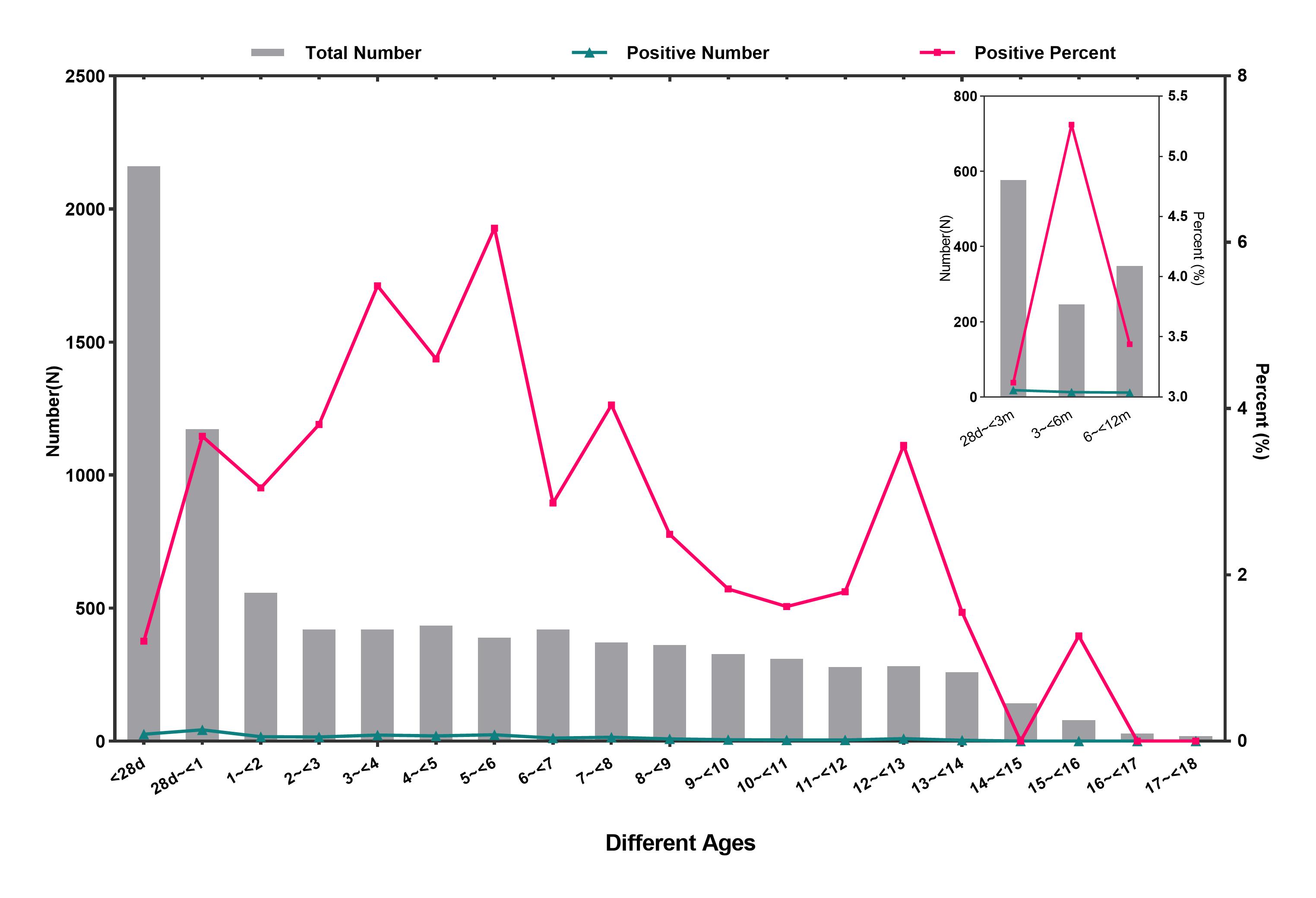
**

**Figure S1. Age distribution of EV-positive patients (n=236).**

**
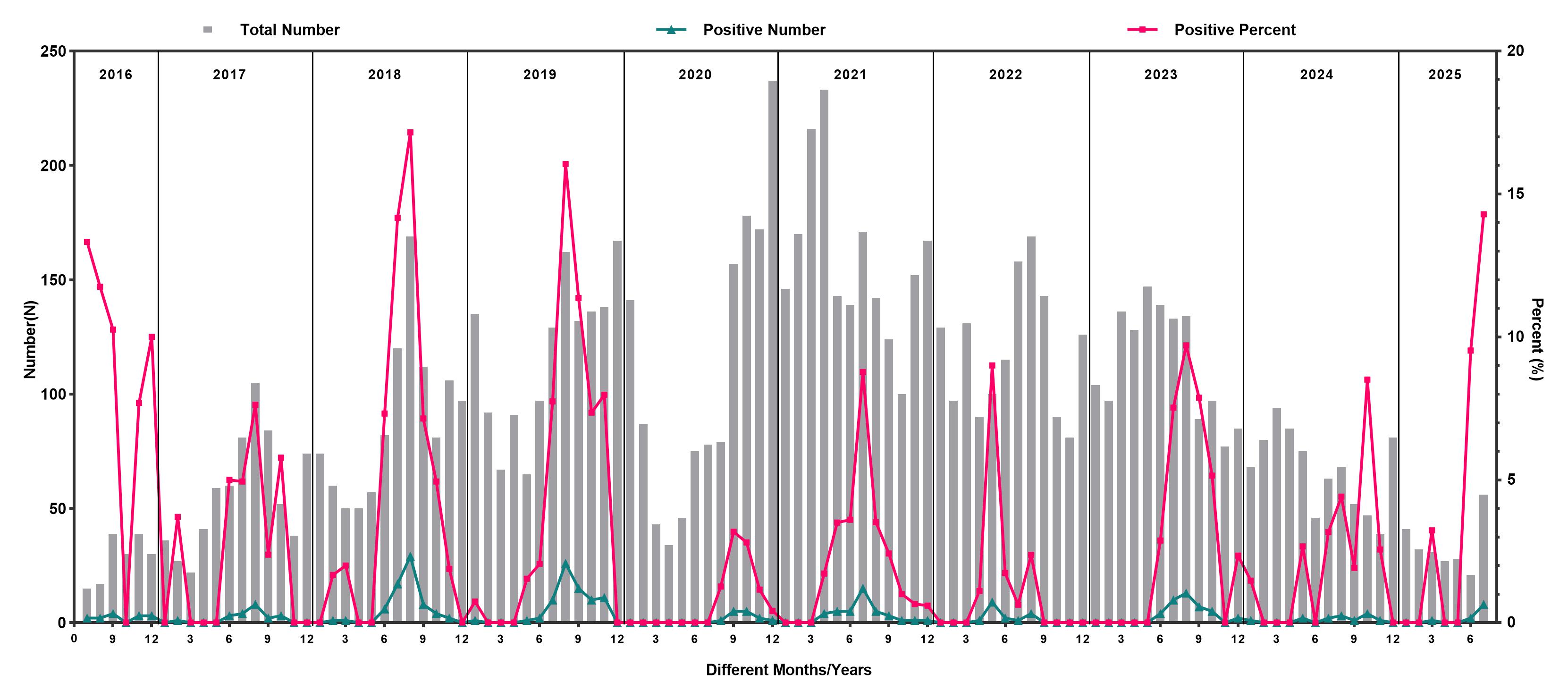
**

**Figure S2. Distribution of** **EV-positive samples** **from 2016 to 2025.** A total of 10,377 samples were included, the detection rate of EVs was 3.43% (316/10,377).

**Table S1 Detection of** **different Samples in different season. (N, %)**

| Seasons | Samples Types | | | |
| --- | --- | --- | --- | --- |
|  | Rectal swab | Throat swab | Cerebrospinal fluid | Plasma |
| Spring | 4(3.20) | 8(2.73) | 4(0.37) | 4(0.51) |
| Summer | 29(16.76) | 31(14.42) | 76(5.67) | 48(4.38) |
| Autumn | 15(6.64) | 24(4.75) | 30(1.94) | 28(2.07) |
| Winter | 0(0.00) | 2(0.61) | 2(0.29) | 11(2.00) |
| Total | 48(8.12) | 65(4.84) | 112(2.41) | 91(2.40) |

**Table S2. Multivariate Logistic Regression Analysis of Risk Factors for EV Infection**

| **Variable** | **Coefficient (β)** | **Standard Error** | **OR Value** | **95% CI** | **P-value** |
| --- | --- | --- | --- | --- | --- |
| Age (vs <28 days) |  |  |  |  |  |
| 28 days to <3 months | 0.2924 | 0.0851 | 2.964 | (1.594-5.510) | 0.001 |
| 3 months to <6 months | 0.1934 | 0.0712 | 3.147 | (1.376-7.200) | 0.007 |
| 6 months to <12 months | 0.2233 | 0.0753 | 3.066 | (1.463-6.428) | 0.003 |
| 1 to <3 years | 0.3549 | 0.0928 | 3.028 | (1.716-5.344) | 0.0001 |
| 3 to <7 years | 0.5803 | 0.1003 | 4.297 | (2.622-7.041) | 0 |
| 7 to <18 years | 0.3364 | 0.1187 | 2.097 | (1.256-3.501) | 0.005 |
| Gender | 0.2071 | 0.0743 | 1.523 | (1.133-2.046) | 0.005 |
| Month | 0.4214 | 0.0757 | 1.128 | (1.081-1.177) | 0 |
